# Genospecies of *Borrelia burgdorferi* sensu lato detected in 16 mammal species and questing ticks from northern Europe

**DOI:** 10.1038/s41598-019-41686-0

**Published:** 2019-03-25

**Authors:** Atle Mysterud, Vetle M. Stigum, Ryanne I. Jaarsma, Hein Sprong

**Affiliations:** 1 Centre for Ecological and Evolutionary Synthesis (CEES), Department of Biosciences, University of Oslo, P.O. Box 1066, Blindern, NO-0316 Oslo Norway; 2 Evolutionary Ecology Group, Department of Biology, University of Antwerp, Universiteitsplein 1, 2610 Wilrijk, Belgium; 3 Centre for Infectious Disease Control (CIb), National Institute for Public Health and the Environment (RIVM), Bilthoven, The Netherlands

## Abstract

Lyme borreliosis is the most common vector-borne zoonosis in the northern hemisphere, and the pathogens causing Lyme borreliosis have distinct, incompletely described transmission cycles involving multiple host groups. The mammal community in Fennoscandia differs from continental Europe, and we have limited data on potential competent and incompetent hosts of the different genospecies of *Borrelia burgdorferi* sensu lato (sl) at the northern distribution ranges where Lyme borreliosis is emerging. We used qPCR to determine presence of *B*. *burgdorferi* sl in tissue samples (ear) from 16 mammalian species and questing ticks from Norway, and we sequenced the 5S–23 S rDNA intergenic spacer region to determine genospecies from 1449 qPCR-positive isolates obtaining 423 sequences. All infections coming from small rodents and shrews were linked to the genospecies *B*. *afzelii*, while *B*. *burgdorferi* sensu stricto (ss) was only found in red squirrels (*Sciurus vulgaris*). Red squirrels were also infected with *B*. *afzelii* and *B*. *garinii*. There was no evidence of *B*. *burgdorferi* sl infection in moose (*Alces alces*), red deer (*Cervus elaphus*) or roe deer (*Capreolus capreolus*), confirming the role of cervids as incompetent hosts. In infected questing ticks in the two western counties, *B*. *afzelii* (67% and 75%) dominated over *B*. *garinii* (27% and 21%) and with only a few recorded *B*. *burgdorferi* ss and *B*. *valaisiana*. *B*. *burgdorferi* ss were more common in adult ticks than in nymphs, consistent with a reservoir in squirrels. Our study identifies potential competent hosts for the different genospecies, which is key to understand transmission cycles at high latitudes of Europe.

## Introduction

Understanding the transmission cycles of pathogens circulating in ecosystems is challenging for multi-host systems^1^. A competent host is defined as a host with the ability to transmit parasites or pathogens such that they effectively infect another host or vector^2^. The numerical balance between competent (or transmission/reservoir) hosts versus incompetent hosts is key to determine the disease hazard, as formulated in the biodiversity buffers disease or dilution hypothesis^3–5^. What constitutes an incompetent or competent host differs across pathogens. An important step to understand disease hazard is hence to determine for a given pathogen which hosts are competent and incompetent in different ecosystems. Among the more complicated enzootic transmission cycles are the ones linked to the generalist ticks of the Ixodidae family in the northern hemisphere^6–8^. These generalist ticks transmit a range of pathogens among which the genospecies forming the *Borrelia burgdorferi* sensu lato (sl) complex causing Lyme borreliosis are the most common and widespread.

The general pattern of the transmission cycle of *B*. *burgdorferi* sl is well-known both in North America, Asia and Europe^6–9^. The number of nymphs infected with *B*. *burgdorferi* sl depends on how many tick larvae get their first blood meal on an infected small vertebrate host^10^. Co-feeding transmission is less important for this group of pathogens, though considerable more for other pathogens like tick-borne encephalitis virus^11^. However, beyond this commonality, there is much specificity in terms of dominating hosts both across and within continents^12^. The *B*. *burgdorferi* sensu stricto (ss) pathogen probably migrated from Europe to North America some 60000 years ago^13^ and this genospecies dominates in North America and evolved into strains infecting birds and small mammals^7^. In Europe, the main pathogenic genospecies to humans are *B*. *afzelii* linked mainly to small rodents, and the *B*. *garinii* linked mainly to birds, while *B*. *burgdorferi* ss is less common and has been linked to red squirrels (*Sciurus vulgaris*) in Switzerland^14^, France^15^ and gray squirrels (*Sciurus carolinensis*) in the UK^16^. However, the competent hosts of the genospecies are partly overlapping and not fully described. Evidence is accumulating that the different genospecies cause different clinical symptoms in humans^17–21^. It is therefore important to understand the transmission dynamics of each of the different genospecies in different regions.

Ticks are expanding their geographical distribution in northern Europe^22,23^, and Lyme disease incidence is documented to increase in both Norway^24,25^ and Finland^26^. The community of mammals in Fennoscandia is different from continental Europe. This is due to both the colder climate and the post-glacial colonization routes both from south and from northeast via the landbridge towards Russia^27^. We have limited data on the pattern of genospecies transmission hosts in these northern ecosystems. We here determined presence of different genospecies of *B*. *burgdorferi* sl in different vertebrate hosts to determine their role as potential transmission host at the northern distribution range of *Ixodes ricinus* in Europe. Furthermore, we determined the relative abundance of *B*. *burgdorferi* sl genospecies in questing ticks as a basis to understand the hazard of each genospecies known to cause different clinical manifestations.

## Results

Infection of *B*. *burgdorferi* sl was found in all rodent and shrew species except in the single house mouse (*Mus musculus*) found (Table 1). With the exception of red squirrels, all sequences of the IGS in rodents and shrews were consistent with infections of *B*. *afzelii*. In 17 red squirrels, we found high infection rates of *B*. *burgdorferi* sl (88%), of which 9 sequences yielded four *B*. *afzelii*, two *B*. *garinii* and three *B*. *burgdorferi* ss. Infection of *B*. *burgdorferi* sl was also found in red fox (*Vulpes vulpes*) and badger (*Meles meles*), but not in the single hare (*Lepus timidus*) included. Attempts to determine the genospecies in red fox and badger were unsuccessful. No infection of *B*. *burgdorferi* sl was found in 111 moose, 28 roe deer or 141 red deer (Table 1). In the eastern region, the infection levels were higher in squirrels (Z = 4.060, P < 0.001) and lower in the Eurasian pygmy shrew (*Sorex minutus*) (Z = −2.922, P = 0.003) compared to the common shrew (*S*. *araneus*), the latter had similar infection levels as the bank vole (*Myodes glareolus*) (Z = 0.206, P = 0.837) and the wood mice (*Apodemus sylvaticus*) (Z = 0.184, P = 0.854).

**Table 1:** The sample sizes (n) and infection prevalence levels in mammals collected from eastern, southern and western Norway.

Common name	Latin name	Region	n	*B*. *burgdorferi* sl			Number of *IGS* sequences		
				Neg	Pos	Prev	*B*. *afzelii*	*B*. *garinii*	*B*. *burgdorferi* ss
Yellow-necked mouse	*Apodemus flavicollis*	SF	19	18	1	0.05			
Wood mouse	*Apodemus sylvaticus*	SF	76	68	8	0.11			
		East	96	71	25	0.26	1		
Field vole	*Microtus agrestis*	SF	41	30	11	0.27	6		
House mouse	*Mus musculus*	SF	1	1	0	0			
Bank vole	*Myodes glareolus*	SF	64	56	8	0.13	1		
		East	333	247	86	0.26	23		
Water shrew	*Neomys fodiens*	SF	15	14	1	0.07			
		East	1	1	0	0			
Common shrew	*Sorex araneus*	SF	554	519	35	0.06	12		
		East	271	203	68	0.25	17		
Taiga shrew	*Sorex isodon*	SF	157	154	3	0.02	1		
Eurasian pygmy shrew	*Sorex minutus*	SF	55	55	0	0			
		East	53	51	2	0.04			
Red squirrel	*Sciurus vulgaris*	East	17	2	15	0.88	4	2	3
Red fox	*Vulpes vulpes*	East	6	5	1	0.17			
Badger	*Meles meles*	East	14	12	2	0.14			
Hare	*Lepus timidus*	East	1	1	0	0			
Roe deer	*Capreolus capreolus*	East	28	28	0	0			
Red deer	*Cervus elaphus*	SF	126	126	0	0			
		South	15	15	0	0			
Moose	*Alces alces*	South	111	111	0	0			

**Figure 1:**
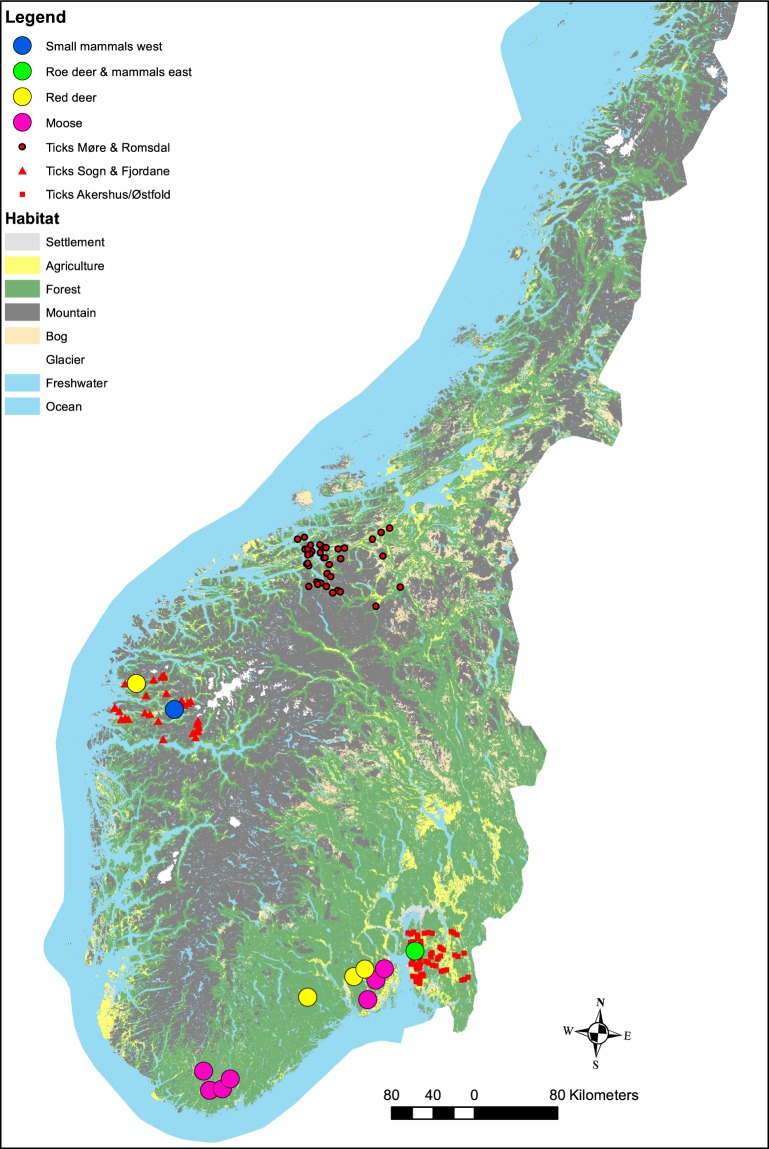
A map of southern Norway with origin of samples of mammal tissue and questing ticks. The ‘roe deer & mammal east’ site include red fox, badger, hare and red squirrel in addition to small rodents and shrews, while ‘small mammals west’ includes small rodents and shrews (Table 1).

Neg/Pos: negative/positive for *B*. *burgdorferi* sl. Prev: prevalence. SF: Sogn and Fjordane county. East: Østfold and Akershus county. South: Telemark and Vestfold county. Locations are given in Fig. 1.

In questing nymphal ticks with infection of *B*. *burgdorferi* sl (Table 2), the genospecies *B*. *afzelii* dominated both in the Sogn & Fjordane (66.7%) and Møre & Romsdal (75.2%) county in the western region, with *B*. *garinii* being common in both Sogn & Fjordane (26.5%) and Møre & Romsdal (21.0%), while *B*. *valaisiana* was less commonly found (Sogn & Fjordane: 6.1%, Møre & Romsdal: 3.8%). Sample sizes for the eastern region was low, and yielded only *B*. *afzelii* in nymphs (n = 8). The same main geographic pattern in terms of genospecies distribution was found in adult male and female ticks. The exception was *B*. *burgdorferi* ss that were found in 6 adult ticks and one nymphal tick overall, and a logistic regression confirmed a higher frequency of *B*. *burgdorferi* ss in adults than nymphs compared to *B*. *afzelii* (Z = −2.071, P = 0.044).

**Table 2:** An overview of *B*. *burgdorferi* sl genospecies in infected questing *I*. *ricinus* ticks in % (n) based on the IGS sequences from Sogn & Fjordane (SF), Møre & Romsdal (MR) and Akershus/Østfold (east) counties of Norway. n-tot = total number of questing ticks; n-pos = number of *B*. *burgdorferi* sl-positive ticks; n-seq = number of positive samples that was successfully sequenced.

Life stage	Region	n-tot	n-pos	n-seq	*B. afzelii*	*B. garinii*	*B. burgdorferi* ss	*B. valaisiana*
Nymphs	SF	4857	567	147	66.7% (98)	26.5% (39)	0.7% (1)	6.1% (9)
	MR	1771	245	105	75.2% (79)	21.0% (22)	0	3.8% (4)
	East	872	99	8	100% (8)	0	0	0
Adult males	SF	626	74	15	66.7% (10)	20.0% (3)	6.7% (1)	6.7% (1)
	MR	264	35	16	81.3% (13)	12.5% (2)	6.3% (1)	0
	East	109	16	0				
Adult females	SF	624	91	35	65.7% (23)	20.0% (7)	5.7% (2)	8.6% (3)
	MR	223	34	17	70.6% (12)	23.5% (4)	0	5.9% (1)
	East	91	22	10	40.0% (4)	40.0% (4)	20.0% (2)	0

To ensure that the *B*. *garinii* classification based on sequencing of 5S–23S rDNA intergenic spacer region (IGS) from positive samples was correct and not included *B*. *bavariensis* (see Methods), we also ran another genetic marker, namely *pepX*, on the 83 positive samples for *B*. *garinii*, of which 51 yielded successful sequences. We confirmed *B*. *garinii* in 49 of the 51 samples, while two cases (one red squirrel in Akershus, one tick nymph in Sogn & Fjordane) came out as *B*. *afzelii*, likely due to infection with both *B*. *garinii* and *B*. *afzelii* (see Discussion).

## Discussion

Determining the competence of vertebrate hosts to pathogens is one of several keys necessary to estimation of disease hazard. A competent host is defined by the ability to be a source of infection to ticks^14,28^. A competent host must be fed on by infected ticks and take up a critical number of the pathogen, and it must allow the pathogen to multiply and to pass on infection to new ticks^28^. Hence, establishing infection is necessary, but on its own does not prove amplification of a pathogen in a host or transmission to a vector. In some cases, DNA from *B*. *burgdorferi* sl can be found without evidence of amplification or transmission to vector ticks^29^, and PCR cannot separate live and dead pathogen DNA. Several other technical issues, such as the sensitivity and specificity of the PCR, tissue tropisms of the pathogen and timing of host collection can also impede the identification of competent hosts. Although the absence of infection can confirm a vertebrate as an incompetent host, lack of pathogen detection by PCR due to technical limitations, cannot firmly establish this. Ideally, laboratory-based transmission experiments^30,31^, as well as field studies focusing on vertebrate communities^32^, are both necessary and complimentary approaches to acquire full understanding of the transmission dynamics of tick-borne pathogens. Our field study hence provides one step towards gaining the required information to identify important potential competent and incompetent hosts at northern latitudes of Europe.

### A new potential competent host of *B*. *afzelii*

Due to its medical importance and relevance of determining disease hazard, there are a huge number of studies reporting frequency of genospecies of *B*. *burgdorferi* sl in Europe. The dominating genospecies in questing ticks is the genospecies *B*. *afzelii* almost universally across continental Europe^33^, while *B*. *garinii* dominates in the UK^8^. Our estimates of 75% and 67% *B*. *afzelii* in the two western counties are close to previous estimates from Norway both in south with 62%^34^, 86% in east and south^35^, and 68% further north of the western study site reported here^36^. This main picture was also found in Sweden^37^. The *B*. *garinii* is typically the second most common genospecies mainly linked to birds^38,39^. Our estimated prevalence in *B*. *burgdorferi* sl infected ticks of 21% and 26% *B*. *garinii* in the two western counties are also close to previous estimates of 23.4% *B*. *garinii* in south^34^, 12% in east and south^35^ and 20.8% in west^36^ of Norway. Further, as reported in these studies, we also found a low prevalence of the bird-borne, but less pathogenic, *B*. *valaisiana*.

The commonness of *B*. *afzelii* in ticks is linked to the high abundance and spread of small mammals, which is its main reservoir. As commonly reported, the bank vole and the wood mice are dominant hosts of larvae and have often high infection levels with *B*. *afzelii*. Wood mice and yellow-necked mice are also considered to be important hosts of *B*. *burgdorferi* sl in urban environments^40^. As we reported earlier^41^, the common shrew is abundant and important in feeding tick larvae in Norway, as also found in Scotland^42^. Based on this study, we can add the taiga shrew (*S*. *isodon*) to the list of potential competent hosts for *B*. *afzelii* in Europe. The taiga shrew has larval tick loads similar to the common shrew (own unpublished data), but prevalence of *B*. *burgdorferi* sl was lower (Table 1). This is a rare species in Norway and Red Listed as ‘data deficient’. However, the species is common in Finland^43^ and further east in Russia. Therefore, the host species involved in transmission may differ in the north relative to continental Europe. There is also a high level of endemism in the Mediterranean small mammals^44^, suggesting that species involved in circulation of *B*. *afzelii* differs with latitude.

### Medium-sized hosts infections

Squirrels are sufficiently large in size to be commonly bitten by nymphal ticks^14–16^, which is required to obtain infections of *B*. *burgdorferi* sl^8^. Their movements on the ground make them regularly exposed to ticks. We indeed found a high infection prevalence in red squirrels. Interestingly, we found infection of three genospecies in red squirrels: *B*. *afzelii*, *B*. *burgdorferi* ss, and *B*. *garinii*. Squirrels seem to regularly be infected by several genospecies of *B*. *burgdorferi* sl. In Switzerland, a mixture of *B*. *afzelii* and *B*. *burgdorferi* ss was found in red squirrels^14^. In France, prevalence of 18.9% *B*. *burgdorferi* s.s., 11.9% *B*. *afzelii*, and 3.5% *B*. *garinii* was found in red squirrels^15^. All four genospecies (*B*. *afzelii*, *B*. *burgdorferi* ss, *B*. *garinii*, *B*. *valaisiana*) that occur in the United Kingdom were detected in gray squirrels, and the commonly bird-associated *B*. *garinii* was most common in the gray squirrels^16^. Our few reports of *B*. *burgdorferi* ss in questing ticks, and mostly in adult ticks, are consistent with a reservoir like squirrels typically having nymphs attached and which are less abundant than small mammals. Another study at the west coast of Norway also found *B*. *burgdorferi* ss more commonly in adult than nymphal ticks^36^.

Infection with several genospecies seem common in medium-sized mammals. More *B*. *burgdorferi* sl genospecies were found in Siberian chipmunks (*Tamias sibericus*) than in the native bank vole^45^. We only retrieved one hare and it was without infection, but infection of both *B*. *afzelii* and *B*. *burgdorferi* ss in hare was documented in south Norway^46^. Hares were also infected with *B*. *garinii* in Sweden^37^. We also documented infection in badger and red fox, but prevalence was low (Table 1). Infection of *B*. *burgdorferi* sl has previously been found in red fox^47^, and badger was reported infected with both *B*. *afzelii* and *B*. *valaisiana*^48^. Medium-sized mammals may hence potentially play roles for transmission of multiple genospecies. Hedgehogs are also important for circulation of *B*. *bavariensis*^49^, and that this genospecies have not been found in Norway may be due to the low population numbers of hedgehogs.

### Amplification and the challenge of co-infections

A quite high proportion of our positive *B*. *burgdorferi* sl samples did not amplify (Tables 1 and 2), which is a requirement in our approach for determining genospecies. The sequencing success decreases with increasing Ct values (i.e., the number of PCR cycles before getting a positive signal). However, only 0.35% (6 of our 865 samples) had Ct values above 40. We cannot identify the cause of lack of amplification with certainty. The most straightforward explanation is that our qPCR-based assay is more sensitive than the conventional PCR^50^. It is also possible that infections with multiple *B*. *burgdorferi* sl genospecies can play a role. Indeed, typing using the pepX marker of our *B*. *garinii*-positive samples based on IGS (see Methods) came up with two *B*. *afzelii* (one in a red squirrel and one in a tick nymph). As *B*. *garinii* and *B*. *afzelii* are readily separated by both methods, this is indicative of co-infection. The aim of our study was not to quantify extent of co-infections, but these kind of co-infections may cause detection bias if amplification success by PCR differs between genospecies, or when *B*. *burgdorferi* sl genospecies have differences in tissue tropism. That we in the small sample of squirrels identified all 3 genospecies using IGS alone would suggest this was not a strong bias. It is therefore likely a robust result that only *B*. *afzelii* was detected in smaller mammals with a much larger sample size, and that this was not due to failure of *B*. *burgdorferi* ss to amplify. Multiple infections in medium-sized hosts may provide a platform for genetic re-arrangements between genospecies, becoming a potential ‘melting-pot’^51^. It can also cause co-infection of genotypes of *B*. *burgdorferi* sl in ticks already at the nymphal stage.

### Cervids as incompetent hosts

A controversial issue is the extent to which biodiversity dilutes disease in general, and how important incompetent hosts are for Lyme borreliosis hazard^52,53^. Several lines of evidence have been used to infer that cervids most likely are incompetent hosts in the enzootic cycle of *B*. *burgdorferi* sl. Commonly, ticks engorged on cervids are used as evidence^54^. However, ticks removed from cervids often contain a low level of infection with *B*. *burgdorferi* sl^55^, which may be due to a number of different mechanisms. In our sizeable sample, the complete lack of detection suggest using ear skin infections are likely better in substantiating that cervids are incompetent hosts than using ticks feeding on cervids. The absence of infection are evidence that cervids are incompetent hosts, but not sufficient evidence that they are dilution hosts. The mechanism of the dilution hypothesis is that when competent hosts are outnumbered by incompetent hosts, the probability of vectors feeding on competent hosts is reduced and so the abundance of infected vectors is lowered^52^. The degree of dilution by cervids depends not only on the relative proportion of the immature tick population fed relative to competent host groups, but also on the absolute number of ticks fed with a change in host population density^52^. High deer density was linked to increased incidence of Lyme disease in Norway^24^, which document that deer mainly act as an amplification (or reproduction) host of tick populations rather than as a dilution host that interferes with the *Borrelia* transmission cycle.

Our study identifies potential competent (or reservoir) and incompetent hosts towards the northern distribution range of ticks in Europe with a different mammal community compared to continental Europe. Future studies are necessary to determine that they are indeed reservoir competent, vector competent and whether the presence of competent and incompetent hosts has a substantial effect on the hazard (density of questing infected nymphs), which will depend on a number of factors including the population densities of the different species involved^52^.

## Material and Methods

### Study areas

The study areas are spread across the southern part of Norway (Fig. 1). The western coast has a warmer and rainier climate compared to eastern areas of Norway. The location of samples and the main habitat types are given in Fig. 1. A more detailed description of the main habitat types can be found in ref. ^24^.

### Small mammal trapping

Small mammals were captured in spring and fall during 2014–16 in Vestby municipality, Akershus county and 2013–16 in Førde and Askvoll municipalities, Sogn & Fjordane county, Norway. Small mammals captured were sacrificed (cervical dislocation) and transferred to an individual zip-lock plastic bag, marked with station number, trap number and date. All bags were stored in a freezer for later observation. The temporal and regional variation in tick load and infection prevalence of *B*. *burgdorferi* sl will be presented elsewhere (own unpublished results).

### Medium and large sized mammals

We used different methods to sample as many different species of mammals as possible. In Vestby municipality in Akershus county and surrounding areas, we sampled road-kills of roe deer, badger, fox, red squirrels and hare from spring 2016. Ears from red deer and moose were sampled in connection with ongoing surveillance for Chronic Wasting Disease fall 2016. For these data, hunters provided full heads to the Norwegian Food Safety Authority, which removed an ear that was then frozen and later transported to CEES, University of Oslo. We obtained further red squirrels from hunting (August 2016).

### Ethics statement

Permissions to capture rodents and shrews and hunt red squirrels outside of hunting season were given by the Norwegian Environment Agency. Small mammals captured were sacrificed on site (see above). The licence by Norwegian Environment Agency is the only requirement to conform to the Norwegian laws and regulations.

### Questing ticks

Questing ticks were sampled regularly along 34 transects in the Sogn & Fjordane county and along 42 transects and 10 other plots in Møre & Romsdal county, western Norway^56,57^. The temporal and regional variation in prevalence of *B*. *burgdorferi* sl in questing nymphs has been presented elsewhere^24,58^. The questing ticks were all identified to *I*. *rinicus* based on morphology.

### DNA extraction

Tissue samples from mammals were derived from the ears, sometimes with surrounding skin for the smallest shrew species. The DNA was extracted with Qiagen blood and tissue kit according to the manufactures recommendations. To easier prepare a high number of samples that later could be extracted, we froze the samples after the overnight incubation with ATL buffer and proteinase K. A total of 94 samples were extracted at the time, leaving two spaces empty for controls. The DNA was stored at −80 °C till later use.

### qPCR protocol

The extracted DNA were screened for *B*. *burgdorferi* sl by realtime PCR (qPCR) in a duplex^59^ (with *A*. *phagocytophilum*, not reported here), at CEES, UiO as in previous work^24,60^, following Allender *et al*.^61^. We used the forward primer CGAGTCTTAAAAGGGCGATTTAGT, the reverse primer GCTTCAGCCTGGCCATAAATAG and the probe [6FAM]AGATGTGGTAGACCCGAAGCCGAGTG[TAMRA] to target the *23 S rRNA* gene of *B*. *burgdorferi* with the fluorescent colour FAM. qPCR reactions were done with a total volume of 10 µl, using 1 µl of DNA and 9 µl of mastermix. A 96 well plate was filled with 94 samples, one positive control and one negative control. A two step program was used on LightCycler 96. Starting with pre incubation of 600 s of 95 °C followed by 50 cycles of two step amplification with 15 s of 95 °C and 60 s of 60 °C.

### Sequencing of 5S–23S rDNA intergenic spacer region (IGS) from positive samples

The DNA from the samples that came up positive from the qPCR, were amplified by conventional PCR, targeting the5S-23S ribosomal RNA intergenic spacer region (*IGS)* of *B*. *burgdorferi*. We used the forward primer B5Sborseq (5′-GAGTTCGCGGGAGAGTAGGTTATTGCC-3′) and the reverse primer 23Sborseq (5′-TCAGGGTACTTAGATGGTTCACTTCC-3′).

To ensure that a product from the PCR was obtained, gel electrophoreses was utilized with 8 µl of DNA on a 1.5% agarose gel. The gel was colored with SYBR™ Gold Nucleic Acid Gel Stain (Invitrogen™). If the PCR was successful showing a clear band on the gel, the DNA was cleaned with ExoSAP-IT™ PCR Product Cleanup Reagent (Applied Biosystems™) and sent to sequencing by the firm BaseClear. The chromatographs of the sequences were visually inspected and the primers sites were trimmed in Bionumerics. Our sequences were used to identify the *B*. *burgdorferi* sl genospecies by comparison to sequences of known genospecies from GenBank (See Supplementary Table 1). The cluster analyses were performed in Bionumerics 7.4 (Applied Math, Belgium) as described previously^62^.

### Sequencing of pepX of *B*. *garinii* positive samples

As it is not always simple to discriminate between *B*. *bavariensis* and *B*. *garinii* based on IGS data, the nested PCR pepX was done for confirmation following Margos *et al*.^63^.

We used the forward primer pepXF362 (5′-ACAGAGACTTAAGCTTAGCAG-3′) and reverse primer pepXR1172 (5′-GTTCCAATGTCAATAGTTTC-3′) for the initial PCR, and added 1 µl of this product to the nested PCR. The nested primers are the forward primer pepXF449 (5′-TTATTCCAAACCTTGCAATCC-3′) and reverse primer pepXR1115 (5′-TGTGCCTGAAGGAACATTTG-3′).

A total of 10 µl of the nested product was analyzed using the QIAxcel DNA High Resolution Kit on the QIAxcel Advanced System (Qiagen, Hilden, Germany 2018), and samples with a clear band were sent to sequencing by the firm BaseClear. The primer sites of our sequences were manually trimmed and processed. The sequences from this study were used to identify the *B*. *burgdorferi* sl genospecies by genetic comparison to sequences of established isolates, as described previously^62^. PepX sequences of known *B*. *burgdorferi* sl genospecies can be retrieved from GenBank or from a *Borrelia* MLST Database (https://pubmlst.org/borrelia/).

### Statistical analysis

We used logistic regression in R vs. 3.4.4 to analyse variation in prevalence of *B*. *burgdorferi* sl with mammal species as a factor. However, the presence of species was not balanced across counties, so that we cannot run a full model. For the most common small mammals, we were mainly interested in comparing infection levels relative to medium-sized mammals. Due to the issue of perfect separation for some species (no positives in cervids), we initially applied a one-step-estimator (maxit = 1), but failed to get convergence. We therefore ended up with an analysis restricted to the eastern region and including rodents and shrews, except water shrew due to low sample size. For questing ticks, we ran a logistic regression to test if genospecies *B*. *afzelii* and *B*. *burgdorferi* ss in questing ticks was found equally often in nymphs or adults (males and females combined) and with county included as a 3-level factor variable.

## Supplementary information

Supplementary info

## Data Availability

All data are reported in Tables 1 and 2 within the paper. Sequences obtained in this study are in Genbank with accession numbers MK108437 to MK108914.
