# Supplementary material for: Genospecies of Borrelia burgdorferi sensu lato detected in 16 mammal species and questing ticks from northern Europe
Source: Sci Rep. 2019 Mar 25;9:5088. doi: 10.1038/s41598-019-41686-0 (PMC6434031; doi:10.1038/s41598-019-41686-0)
Supplement: Supplementary file 1 — Supplementary info [file 41598_2019_41686_MOESM1_ESM.pdf]

Genospecies of *Borrelia burgdorferi* sensu lato detected in 16 mammal species and questing ticks from northern Europe

Atle Mysterud, Vetle M. Stigum, Ryanne I. Jaarsma, and Hein Sprong

Supplementary Information

Content:

- Supplementary Table 1

Supplementary Table 1. An overview of Genbank Accession Numbers and sequences linked to genospecies used for comparison to determine genospecies in our current sample.

**Genbank Accession number**	**Country**	**Strain information**	**Genospecies**
AB189458	Serbia	Borrelia afzelii DNA, 5S-23S rRNA intergenic spacer, partial sequence.	afzelii
AF090971	Slovakia	Borrelia burgdorferi strain MIL internal transcribed spacer 2, complete seq.	burgdorferi
AF090972	Germany	Borrelia burgdorferi strain Z136 internal transcribed spacer 2, complete seq.	burgdorferi
AF090973	Switzerland	Borrelia burgdorferi strain NE271 internal transcribed spacer 2, complete seq.	burgdorferi
AF090974	Switzerland	Borrelia burgdorferi strain NE99 internal transcribed spacer 2, complete seq.	burgdorferi
AF090975	Denmark	Borrelia burgdorferi strain DK7 internal transcribed spacer 2, complete seq.	burgdorferi
AF090976	Spain	Borrelia burgdorferi strain Esp1 internal transcribed spacer 2, complete seq.	burgdorferi
AF090977	France	Borrelia burgdorferi strain IP1 internal transcribed spacer 2, complete seq.	burgdorferi
AF090978	Italy	Borrelia burgdorferi strain HII internal transcribed spacer 2, complete seq.	burgdorferi
AF090979	Austria	Borrelia burgdorferi strain L5 internal transcribed spacer 2, complete seq.	burgdorferi
AF090980	Germany	Borrelia burgdorferi strain 35B808 internal transcribed spacer 2, complete seq.	burgdorferi
AF090981	Switzerland	Borrelia burgdorferi strain NE56 internal transcribed spacer 2, complete seq.	burgdorferi
AF090982	France	Borrelia burgdorferi strain 20006 internal transcribed spacer 2, complete seq.	burgdorferi
AF090983	Switzerland	Borrelia sp. NE581 internal transcribed spacer 2, complete sequence.	burgdorferi
AF090984	Switzerland	Borrelia sp. NE49 internal transcribed spacer 2, complete sequence.	burgdorferi
AF090985	Russia	Borrelia sp. Ir-3519 internal transcribed spacer 2, complete sequence.	burgdorferi
AF497979	Czech Republic	Borrelia burgdorferi genotype A26 5S-23S ribosomal RNA intergenic spacer region	burgdorferi
AF497980	Czech Republic	Borrelia burgdorferi genotype I-181 5S-23S ribosomal RNA intergenic spacer regi	burgdorferi
AF497981	Czech Republic	Borrelia burgdorferi genotype I-208 5S-23S ribosomal RNA intergenic spacer regi	burgdorferi
AF497982	Czech Republic	Borrelia afzelii genotype D-3 5S-23S ribosomal RNA intergenic spacer region, pa	afzelii
AF497983	Czech Republic	Borrelia afzelii genotype I-2 5S-23S ribosomal RNA intergenic spacer region, pa	afzelii
AF497985	Czech Republic	Borrelia valaisiana genotype D-2 5S-23S ribosomal RNA intergenic spacer region,	valaisiana

AF497986	Czech Republic	Borrelia valaisiana genotype I-52 5S-23S ribosomal RNA intergenic spacer region	valaisiana
AF497987	Czech Republic	Borrelia valaisiana genotype I-30 5S-23S ribosomal RNA intergenic spacer region	valaisiana
AF497988	Czech Republic	Borrelia valaisiana genotype I-214 5S-23S ribosomal RNA intergenic spacer regio	valaisiana
AF497989	Czech Republic	Borrelia valaisiana genotype B-4 5S-23S ribosomal RNA intergenic spacer region,	valaisiana
AF497994	Czech Republic	Borrelia spielmani 5S-23S ribosomal RNA intergenic spacer, partial sequence.	spielmanii
AF538271	Russia	Borrelia afzelii isolate 63 5S-23S ribosomal RNA intergenic spacer, partial seq	afzelii
AM160603	Germany	Borrelia burgdorferi 5S-23S rRNA intergenic spacer, strain PSigII.	spielmanii
AM160604	Germany	Borrelia burgdorferi 5S-23S rRNA intergenic spacer, strain PHap.	spielmanii
AM160605	Germany	Borrelia burgdorferi 5S-23S rRNA intergenic spacer, strain PMew.	spielmanii
AM160606	Germany	Borrelia burgdorferi 5S-23S rRNA intergenic spacer, strain PMai.	spielmanii
AM160607	Slovenia	Borrelia burgdorferi 5S-23S rRNA intergenic spacer, strain PAnz.	spielmanii
AM160608	Slovenia	Borrelia burgdorferi 5S-23S rRNA intergenic spacer, strain PJes.	spielmanii
DQ111065	Portugal	Borrelia lusitaniae strain PotiB1 rrf-rrl intergenic spacer, partial sequence.	lusitaniae
DQ133526	France	Borrelia spielmanii strain PC-Eq17N5 rrf-rrl intergenic spacer, partial sequenc	spielmanii
DQ133527	France	Borrelia spielmanii strain PC-Eq2/1W10 rrf-rrl intergenic spacer, partial seque	spielmanii
DQ133528	France	Borrelia spielmanii strain PC-Eq2rN8 rrf-rrl intergenic spacer, partial sequenc	spielmanii
DQ393307	?	Borrelia burgdorferi strain Sh-2-82 rrf-rrl intergenic spacer, partial sequence	burgdorferi
DQ393308	?	Borrelia burgdorferi strain 5LM218 rrf-rrl intergenic spacer, partial sequence.	burgdorferi
DQ517433	Slovakia	Borrelia garinii strain 6-2N10 5S-23S ribosomal RNA intergenic spacer, partial	garinii
DQ520861	Slovakia	Borrelia garinii strain 6-6L6 5S-23S ribosomal RNA intergenic spacer, partial s	garinii
DQ520862	Slovakia	Borrelia garinii strain 6-6N1 5S-23S ribosomal RNA intergenic spacer, partial s	garinii
DQ520863	Slovakia	Borrelia garinii strain 6-9N2 5S-23S ribosomal RNA intergenic spacer, partial s	garinii
DQ520864	Slovakia	Borrelia garinii strain 6-16L1 5S-23S ribosomal RNA intergenic spacer, partial	garinii
DQ520865	Slovakia	Borrelia garinii strain 6-16N3 5S-23S ribosomal RNA intergenic spacer, partial	garinii
DQ520869	Slovakia	Borrelia garinii strain 248D 5S-23S ribosomal RNA intergenic spacer, partial se	garinii
DQ520870	Slovakia	Borrelia garinii strain 453Y 5S-23S ribosomal RNA intergenic spacer, partial se	garinii
DQ520871	Slovakia	Borrelia garinii strain 6-16N12 5S-23S ribosomal RNA intergenic spacer, partial	garinii
DQ520872	Slovakia	Borrelia garinii strain 6-10N10 5S-23S ribosomal RNA intergenic spacer, partial	garinii
DQ520873	Slovakia	Borrelia garinii strain 398D 5S-23S ribosomal RNA intergenic spacer, partial se	garinii
DQ860257	Italy	Borrelia afzelii isolate TN1 rrf-rrl intergenic spacer and 23S ribosomal RNA ge	afzelii

DQ860258	Italy	Borrelia afzelii isolate TN2 rrf-rrl intergenic spacer and 23S ribosomal RNA ge	afzelii
DQ860259	Italy	Borrelia afzelii isolate TN4 rrf-rrl intergenic spacer and 23S ribosomal RNA ge	afzelii
DQ860260	Italy	Borrelia afzelii isolate TN5 rrf-rrl intergenic spacer and 23S ribosomal RNA ge	afzelii
DQ860262	Italy	Borrelia valaisiana isolate TN7 rrf-rrl intergenic spacer and 23S ribosomal RNA	valaisiana
DQ860263	Italy	Borrelia valaisiana isolate TN8 rrf-rrl intergenic spacer and 23S ribosomal RNA	valaisiana
DQ860264	Italy	Borrelia valaisiana isolate TN9 rrf-rrl intergenic spacer and 23S ribosomal RNA	valaisiana
DQ860265	Italy	Borrelia valaisiana isolate TN10 rrf-rrl intergenic spacer and 23S ribosomal RN	valaisiana
DQ860266	Italy	Borrelia garinii isolate TN12 rrf-rrl intergenic spacer and 23S ribosomal RNA g	garinii
DQ860267	Italy	Borrelia garinii isolate TN14 rrf-rrl intergenic spacer and 23S ribosomal RNA g	garinii
DQ860268	Italy	Borrelia garinii isolate TN16 rrf-rrl intergenic spacer and 23S ribosomal RNA g	garinii
DQ860269	Italy	Borrelia garinii isolate TN17 rrf-rrl intergenic spacer and 23S ribosomal RNA g	garinii
DQ860270	Italy	Borrelia garinii isolate TN18 rrf-rrl intergenic spacer and 23S ribosomal RNA g	garinii
DQ860271	Italy	Borrelia burgdorferi isolate TN19 rrf-rrl intergenic spacer and 23S ribosomal R	burgdorferi
DQ860272	Italy	Borrelia burgdorferi isolate TN20 rrf-rrl intergenic spacer and 23S ribosomal R	burgdorferi
DQ860273	Italy	Borrelia garinii isolate TN11 rrf-rrl intergenic spacer and 23S ribosomal RNA g	garinii
EF179598	Portugal	Borrelia lusitaniae strain PoHL1 5S-23S ribosomal RNA intergenic spacer, partia	lusitaniae
EF488984	Russia	Borrelia garinii strain Nov11106 5S-23S ribosomal RNA intergenic spacer, partia	garinii
EF647595	Portugal	Borrelia lusitaniae strain PoAnB1 rrf-rrl intergenic spacer, partial sequence.	lusitaniae
EU078961	Portugal	Borrelia lusitaniae strain PoTiB6 rrf-rrl intergenic spacer, partial sequence.	lusitaniae
EU401776	Czech Republic	Borrelia garinii isolate TP 394/1 5S-23S ribosomal RNA intergenic spacer, parti	garinii
EU401777	Czech Republic	Borrelia garinii isolate TP 65/1 5S-23S ribosomal RNA intergenic spacer, partia	garinii
EU401778	Czech Republic	Borrelia garinii isolate TP 66/1 5S-23S ribosomal RNA intergenic spacer, partia	garinii
EU401779	Czech Republic	Borrelia garinii isolate PM 72/2 5S-23S ribosomal RNA intergenic spacer, partia	garinii
EU401780	Czech Republic	Borrelia garinii isolate PM 72/4 5S-23S ribosomal RNA intergenic spacer, partia	garinii
EU401781	Czech Republic	Borrelia garinii isolate PM 72/5 5S-23S ribosomal RNA intergenic spacer, partia	garinii
FJ431126	Czech Republic	Borrelia garinii isolate p1BgA 5S-23S ribosomal RNA intergenic spacer, partial	garinii
FJ431127	Czech Republic	Borrelia garinii isolate p20Bg 5S-23S ribosomal RNA intergenic spacer, partial	garinii

FJ431128	Czech Republic	Borrelia burgdorferi isolate p2H12 5S-23S ribosomal RNA intergenic spacer, part	burgdorferi
FJ431129	Czech Republic	Borrelia burgdorferi isolate p1f3 5S-23S ribosomal RNA intergenic spacer, parti	burgdorferi
FJ431130	Czech Republic	Borrelia burgdorferi isolate p5d5 5S-23S ribosomal RNA intergenic spacer, parti	burgdorferi
FJ431131	Czech Republic	Borrelia burgdorferi isolate p6f1 5S-23S ribosomal RNA intergenic spacer, parti	burgdorferi
FJ431132	Czech Republic	Borrelia burgdorferi isolate p8g1 5S-23S ribosomal RNA intergenic spacer, parti	burgdorferi
FJ431133	Czech Republic	Borrelia burgdorferi isolate p9g2 5S-23S ribosomal RNA intergenic spacer, parti	burgdorferi
FJ431134	Czech Republic	Borrelia burgdorferi isolate p15h1 5S-23S ribosomal RNA intergenic spacer, part	burgdorferi
FJ431135	Czech Republic	Borrelia burgdorferi isolate p16d1 5S-23S ribosomal RNA intergenic spacer, part	burgdorferi
FJ431136	Czech Republic	Borrelia bissettii isolate p1c8 5S-23S ribosomal RNA intergenic spacer, partial	bissettii
FJ431137	Czech Republic	Borrelia bissettii isolate p2e4 5S-23S ribosomal RNA intergenic spacer, partial	bissettii
FJ431138	Czech Republic	Borrelia bissettii isolate p3h10 5S-23S ribosomal RNA intergenic spacer, partia	bissettii
FJ431139	Czech Republic	Borrelia bissettii isolate p9b3 5S-23S ribosomal RNA intergenic spacer, partial	bissettii
FJ431140	Czech Republic	Borrelia bissettii isolate p11a8 5S-23S ribosomal RNA intergenic spacer, partia	bissettii
FJ431141	Czech Republic	Borrelia bissettii isolate p13b1 5S-23S ribosomal RNA intergenic spacer, partia	bissettii
FJ431142	Czech Republic	Borrelia bissettii isolate p16a2 5S-23S ribosomal RNA intergenic spacer, partia	bissettii
FJ546482	France	Borrelia afzelii strain IBS11 5S-23S ribosomal RNA intergenic spacer, partial s	afzelii
FJ546483	France	Borrelia afzelii strain IBS12 5S-23S ribosomal RNA intergenic spacer, partial s	afzelii
FJ546484	France	Borrelia afzelii strain IBS13 5S-23S ribosomal RNA intergenic spacer, partial s	afzelii
FJ546485	France	Borrelia afzelii strain IPT109 5S-23S ribosomal RNA intergenic spacer, partial	afzelii
FJ546486	France	Borrelia afzelii strain IPT110 5S-23S ribosomal RNA intergenic spacer, partial	afzelii
FJ546487	France	Borrelia afzelii strain IPT118 5S-23S ribosomal RNA intergenic spacer, partial	afzelii
FJ546488	France	Borrelia afzelii strain IPT122 5S-23S ribosomal RNA intergenic spacer, partial	afzelii

FJ546489	France	Borrelia afzelii strain IPT138 5S-23S ribosomal RNA intergenic spacer, partial	afzelii
FJ546490	France	Borrelia afzelii strain IPT142 5S-23S ribosomal RNA intergenic spacer, partial	afzelii
FJ546491	France	Borrelia afzelii strain IPT154 5S-23S ribosomal RNA intergenic spacer, partial	afzelii
FJ546492	France	Borrelia afzelii strain IPT164 5S-23S ribosomal RNA intergenic spacer, partial	afzelii
FJ546493	France	Borrelia afzelii strain IPT179 5S-23S ribosomal RNA intergenic spacer, partial	afzelii
FJ546495	Germany	Borrelia bavariensis strain PFlk 5S-23S ribosomal RNA intergenic spacer, partia	bavariensis
FJ546496	Slovenia	Borrelia bavariensis strain PTrob 5S-23S ribosomal RNA intergenic spacer, parti	bavariensis
FJ546497	Austria	Borrelia bavariensis strain PRab 5S-23S ribosomal RNA intergenic spacer, partia	bavariensis
FJ546498	Germany	Borrelia bavariensis strain POb 5S-23S ribosomal RNA intergenic spacer, partial	bavariensis
FJ546499	France	Borrelia garinii strain IPT28 5S-23S ribosomal RNA intergenic spacer, partial s	garinii
FJ546500	France	Borrelia garinii strain IPT114 5S-23S ribosomal RNA intergenic spacer, partial	garinii
FJ546501	France	Borrelia garinii strain IPT130 5S-23S ribosomal RNA intergenic spacer, partial	garinii
FJ546502	France	Borrelia garinii strain IPT139 5S-23S ribosomal RNA intergenic spacer, partial	garinii
FJ546503	France	Borrelia garinii strain IPT140 5S-23S ribosomal RNA intergenic spacer, partial	garinii
FJ546504	France	Borrelia garinii strain IPT156 5S-23S ribosomal RNA intergenic spacer, partial	garinii
FJ546505	France	Borrelia garinii strain IPT157 5S-23S ribosomal RNA intergenic spacer, partial	garinii
FJ546506	France	Borrelia garinii strain IPT158 5S-23S ribosomal RNA intergenic spacer, partial	garinii
FJ546507	France	Borrelia garinii strain IPT165 5S-23S ribosomal RNA intergenic spacer, partial	garinii
FJ546508	France	Borrelia garinii strain IPT167 5S-23S ribosomal RNA intergenic spacer, partial	garinii
FJ546509	France	Borrelia garinii strain IPT168 5S-23S ribosomal RNA intergenic spacer, partial	garinii
FJ546510	France	Borrelia garinii strain IPT169 5S-23S ribosomal RNA intergenic spacer, partial	garinii
FJ546511	France	Borrelia garinii strain IPT171 5S-23S ribosomal RNA intergenic spacer, partial	garinii
FJ546512	France	Borrelia garinii strain IPT172 5S-23S ribosomal RNA intergenic spacer, partial	garinii
FJ546513	France	Borrelia garinii strain IPT178 5S-23S ribosomal RNA intergenic spacer, partial	garinii
FJ546514	France	Borrelia garinii strain IPT189 5S-23S ribosomal RNA intergenic spacer, partial	garinii
FJ546515	France	Borrelia valaisiana strain IPT29 5S-23S ribosomal RNA intergenic spacer, partia	valaisiana
FJ546516	France	Borrelia valaisiana strain IPT31 5S-23S ribosomal RNA intergenic spacer, partia	valaisiana
FJ546517	France	Borrelia valaisiana strain IPT33 5S-23S ribosomal RNA intergenic spacer, partia	valaisiana
FJ546518	France	Borrelia valaisiana strain IPT47 5S-23S ribosomal RNA intergenic spacer, partia	valaisiana
FJ546519	France	Borrelia valaisiana strain IPT85 5S-23S ribosomal RNA intergenic spacer, partia	valaisiana
FJ546520	France	Borrelia valaisiana strain IPT102 5S-23S ribosomal RNA intergenic spacer, parti	valaisiana
FJ546521	France	Borrelia valaisiana strain IPT111 5S-23S ribosomal RNA intergenic spacer, parti	valaisiana
FJ546522	France	Borrelia valaisiana strain IPT121 5S-23S ribosomal RNA intergenic spacer, parti	valaisiana
FJ546523	France	Borrelia valaisiana strain IPT144 5S-23S ribosomal RNA intergenic spacer, parti	valaisiana

FJ546524	France	Borrelia valaisiana strain IPT163 5S-23S ribosomal RNA intergenic spacer, parti	valaisiana
FJ546525	France	Borrelia valaisiana strain IPT166 5S-23S ribosomal RNA intergenic spacer, parti	valaisiana
FJ546526	France	Borrelia valaisiana strain IPT174 5S-23S ribosomal RNA intergenic spacer, parti	valaisiana
FJ546527	France	Borrelia valaisiana strain IPT177 5S-23S ribosomal RNA intergenic spacer, parti	valaisiana
FJ546528	France	Borrelia valaisiana strain IPT184 5S-23S ribosomal RNA intergenic spacer, parti	valaisiana
FJ546529	France	Borrelia valaisiana strain IPT186 5S-23S ribosomal RNA intergenic spacer, parti	valaisiana
FJ546530	France	Borrelia valaisiana strain IPT187 5S-23S ribosomal RNA intergenic spacer, parti	valaisiana
FJ546531	France	Borrelia valaisiana strain IPT188 5S-23S ribosomal RNA intergenic spacer, parti	valaisiana
FJ546532	France	Borrelia burgdorferi strain IPT2 5S-23S ribosomal RNA intergenic spacer, partia	burgdorferi
FJ546533	France	Borrelia burgdorferi strain IPT19 5S-23S ribosomal RNA intergenic spacer, parti	burgdorferi
FJ546534	France	Borrelia burgdorferi strain IPT23 5S-23S ribosomal RNA intergenic spacer, parti	burgdorferi
FJ546535	France	Borrelia burgdorferi strain IPT39 5S-23S ribosomal RNA intergenic spacer, parti	burgdorferi
FJ546536	France	Borrelia burgdorferi strain IPT58 5S-23S ribosomal RNA intergenic spacer, parti	burgdorferi
FJ546537	France	Borrelia burgdorferi strain IPT69 5S-23S ribosomal RNA intergenic spacer, parti	burgdorferi
FJ546538	France	Borrelia burgdorferi strain IPT135 5S-23S ribosomal RNA intergenic spacer, part	burgdorferi
FJ546539	France	Borrelia burgdorferi strain IPT137 5S-23S ribosomal RNA intergenic spacer, part	burgdorferi
FJ546540	France	Borrelia burgdorferi strain IPT190 5S-23S ribosomal RNA intergenic spacer, part	burgdorferi
FJ546541	France	Borrelia burgdorferi strain IPT191 5S-23S ribosomal RNA intergenic spacer, part	burgdorferi
FJ546542	France	Borrelia burgdorferi strain IPT193 5S-23S ribosomal RNA intergenic spacer, part	burgdorferi
FJ546543	France	Borrelia garinii strain IPT195 5S-23S ribosomal RNA intergenic spacer, partial	garinii
FJ546544	France	Borrelia burgdorferi strain IPT198 5S-23S ribosomal RNA intergenic spacer, part	burgdorferi
FJ546545	Switzerland	Borrelia burgdorferi strain NE49 5S-23S ribosomal RNA intergenic spacer, partia	burgdorferi
FJ546546	Germany	Borrelia burgdorferi strain Z41293 5S-23S ribosomal RNA intergenic spacer, part	burgdorferi
FJ546547	Germany	Borrelia burgdorferi strain Z41493 5S-23S ribosomal RNA intergenic spacer, part	burgdorferi
FJ810217	china	Borrelia afzelii strain JX1 5S-23S ribosomal RNA intergenic spacer, partial seq	afzelii
FJ976200	Russia	Borrelia afzelii strain NU06Rut05 5S-23S ribosomal RNA intergenic spacer, parti	afzelii

FJ976201	Russia	Borrelia afzelii strain NU74Glar05 5S-23S ribosomal RNA intergenic spacer, part	afzelii
FN658707	Italy	Borrelia lusitaniae partial 23S-5S rRNA intergenic spacer, isolate IR-PTC 1.	lusitaniae
FN658708	Italy	Borrelia lusitaniae partial 23S-5S rRNA intergenic spacer, isolate IR-PTC 2.	lusitaniae
GQ178225	Germany	Borrelia sp. PFin 5S-23S ribosomal RNA intergenic spacer, partial sequence.	bavariensis
GQ178226	Germany	Borrelia sp. PBN 5S-23S ribosomal RNA intergenic spacer, partial sequence.	bavariensis
GQ178227	Germany	Borrelia sp. PScf 5S-23S ribosomal RNA intergenic spacer, partial sequence.	bavariensis
GQ178229	Germany	Borrelia sp. PBaeI 5S-23S ribosomal RNA intergenic spacer, partial sequence.	bavariensis
GQ387029	Switzerland	Borrelia garinii strain NE11 5S ribosomal RNA gene, partial sequence; 5S-23S ri	garinii
GQ387030	Switzerland	Borrelia garinii strain NEd1 5S ribosomal RNA gene, partial sequence; 5S-23S ri	garinii
GQ387031	Switzerland	Borrelia garinii strain NEd2 5S ribosomal RNA gene, partial sequence; 5S-23S ri	garinii
GQ387032	Switzerland	Borrelia garinii strain NEd3 5S ribosomal RNA gene, partial sequence; 5S-23S ri	garinii
GQ387033	Switzerland	Borrelia garinii strain NEd4 5S ribosomal RNA gene, partial sequence; 5S-23S ri	garinii
GQ903682	Switzerland	Borrelia bavariensis strain NE2802 5S ribosomal RNA gene, partial sequence; 5S-	bavariensis
GQ903683	Switzerland	Borrelia bavariensis strain NE2806 5S ribosomal RNA gene, partial sequence; 5S-	bavariensis
GQ903685	Switzerland	Borrelia garinii strain NE4874 5S ribosomal RNA gene, partial sequence; 5S-23S	garinii
JF331066	united kingdom	Borrelia garinii strain T1131 5S-23S ribosomal RNA intergenic spacer, partial s	garinii
JF331067	united kingdom	Borrelia garinii strain T1377 5S-23S ribosomal RNA intergenic spacer, partial s	garinii
JF331098	Iceland	Borrelia garinii strain T2023 5S-23S ribosomal RNA intergenic spacer, partial s	garinii
JF331099	Iceland	Borrelia garinii strain T2024 5S-23S ribosomal RNA intergenic spacer, partial s	garinii
JF331101	Russia	Borrelia garinii strain T2645 5S-23S ribosomal RNA intergenic spacer, partial s	garinii
JF331102	Russia	Borrelia garinii strain T2712 5S-23S ribosomal RNA intergenic spacer, partial s	garinii
JF331103	Russia	Borrelia garinii strain T2885 5S-23S ribosomal RNA intergenic spacer, partial s	garinii
JF331104	Russia	Borrelia garinii strain T2916 5S-23S ribosomal RNA intergenic spacer, partial s	garinii
JF331105	Russia	Borrelia garinii strain T2929 5S-23S ribosomal RNA intergenic spacer, partial s	garinii
JF331106	Russia	Borrelia garinii strain T2937 5S-23S ribosomal RNA intergenic spacer, partial s	garinii
JF331107	Russia	Borrelia garinii strain T3221 5S-23S ribosomal RNA intergenic spacer, partial s	garinii
JF331108	France	Borrelia garinii strain IPT28 5S-23S ribosomal RNA intergenic spacer, partial s	garinii
JF331109	France	Borrelia garinii strain IPT114 5S-23S ribosomal RNA intergenic spacer, partial	garinii
JF331110	France	Borrelia garinii strain IPT156 5S-23S ribosomal RNA intergenic spacer, partial	garinii
JF331111	France	Borrelia garinii strain IPT167 5S-23S ribosomal RNA intergenic spacer, partial	garinii

JF331112	France	Borrelia garinii strain IPT189 5S-23S ribosomal RNA intergenic spacer, partial	garinii
JF331113	Ireland	Borrelia garinii strain ConnA3/3 5S-23S ribosomal RNA intergenic spacer, partia	garinii
JF331114	Sweden	Borrelia garinii strain G25 5S-23S ribosomal RNA intergenic spacer, partial seq	garinii
JF331117	Switzerland	Borrelia garinii strain CNE 83 5S-23S ribosomal RNA intergenic spacer, partial	garinii
KJ508861	Finland	Borrelia garinii isolate 3,1,1,6 Hrhov 5S-23S intergenic spacer, partial sequen	garinii
KJ508862	Finland	Borrelia garinii isolate 3,1,3,1 Hrhov 5S-23S intergenic spacer, partial sequen	garinii
KJ508863	Finland	Borrelia garinii isolate 5112 Hrhov 5S-23S intergenic spacer, partial sequence.	garinii
KJ508864	Finland	Borrelia garinii isolate 51212 Hrhov 5S-23S intergenic spacer, partial sequence	garinii
KJ508865	Finland	Borrelia garinii isolate 51214 Hrhov 5S-23S intergenic spacer, partial sequence	garinii
KJ577538	Finland	Borrelia garinii strain IO-TP-TW 5S-23S ribosomal RNA intergenic spacer, partia	garinii
L30119	France	Borrelia garinii (strain 20047) internal transcribed spacer.	garinii
L30130	Japan	Borrelia garinii (strain NT29) internal transcribed spacer.	garinii
L30131	Portugal	Borrelia burgdorferi (strain PotiB2) sensu lato internal transcribed spacer.	lusitaniae
L30132	Portugal	Borrelia burgdorferi (strain PotiB3) sensu lato internal transcribed spacer.	lusitaniae
X85745	Italy	B.burgdorferi 23S rRNA, 5S rRNA and 23S rRNA genes.	burgdorferi
Z77166	Germany	B.burgdorferi sensu stricto rrf-rrl internal transcribed spacer DNA (isolate Z7	burgdorferi
Z77167	Germany	B.burgdorferi sensu stricto rrf-rrl internal transcribed spacer DNA (isolate Z5	burgdorferi
Z77168	Germany	B.burgdorferi sensu stricto rrf-rrl internal transcribed spacer DNA (isolate Z5	burgdorferi
Z77169	Germany	B.burgdorferi sensu stricto rrf-rrl internal transcribed spacer DNA (isolate Z5	burgdorferi
Z77170	Germany	B.burgdorferi sensu stricto rrf-rrl internal transcribed spacer DNA (isolate Z4	burgdorferi
Z77171	Germany	B.burgdorferi sensu stricto rrf-rrl internal transcribed spacer DNA (isolate Z5	burgdorferi
Z77172	Germany	B.burgdorferi sensu stricto rrf-rrl internal transcribed spacer DNA (isolate Z4	burgdorferi
Z77176	Germany	B.garinii rrf-rrl internal transcribed spacer DNA (isolate Z61592).	garinii
Z77177	Germany	B.garinii rrf-rrl internal transcribed spacer DNA (isolate Z71194).	garinii
